# Effects of Phenolic Phytogenic Feed Additives on Certain Oxidative Damage Biomarkers and the Performance of Primiparous Sows Exposed to Heat Stress under Field Conditions

**DOI:** 10.3390/antiox11030593

**Published:** 2022-03-20

**Authors:** Vasileios G. Papatsiros, Eleni G. Katsogiannou, Georgios I. Papakonstantinou, Alfred Michel, Konstantinos Petrotos, Labrini V. Athanasiou

**Affiliations:** 1 Clinic of Medicine, Faculty of Veterinary Medicine, School of Health Sciences, University of Thessaly, Trikalon 224, 43100 Karditsa, Greece; elkatsog@uth.gr (E.G.K.); geopapak@vet.uth.gr (G.I.P.); 2 Life Circle Nutrition AG, Hämmerli 2d, 8855 Wangen, Switzerland; a.michel@lifecirclenutrition.com; 3 Department of Agrotechnology, School of Agricultural Sciences, Geopolis Campus, University of Thessaly, Periferiaki Odos Larisas Trikalon, 41500 Larisa, Greece; petrotos@teilar.gr

**Keywords:** phenolic, PFAs, heat stress, TBARS, protein carbonyls, pig

## Abstract

The aim of this study was to investigate the effects of two commercial phenolic phytogenic feed additives (PFAs) on sows under heat stress conditions of high summer temperatures for seven days before and seven days after the farrowing. The PFA-1 product was a mixture based on the plants *Emblica officinalis*, *Foeniculum vulgare*, *Citrus sinensis* and nut fiber, while the PFA-2 product was a mixture based on plants *Andrographis paniculata*, *Glycyrrhizia glabra*, *Tinospora cordifolia* and nut fiber. A total of 48 primiparous sows were divided into three groups: T1-control group: regular gestation (GF) and lactation feed (LF); T2 group: regular GF and LF supplemented with PFA-1; T3 group: regular GF and LF supplemented with PFA-2. Each sow in the T2 and T3 groups received 5 g daily of the PFA-1 and PFA-2 product, respectively, for seven days before and seven days after the farrowing. Blood samples were collected from all groups 24 h after farrowing. Thiobarbituric acid-–reactive substances (TBARS) and protein carbonyl (CARB) concentrations were determined in the sow plasma. The body condition scoring (BCS) and the backfat of sows on the farrowing and weaning days along with reproductive parameters and litter characteristics were recorded. The highest number of stillborn piglets and the largest interval from weaning to estrus were observed in the T1 group. The lowest number of alive 24 h after birth and weaning piglets and the lowest BCS and backfat at weaning were also recorded in the T1 group. TBARS and CARB concentrations were significant higher in the T1 group compared to all other groups. In conclusion, the use of phenolic PFAs seems to reduce oxidative damage caused by heat stress and ameliorate performance in primiparous sows.

## 1. Introduction

Nowadays, climate change is denoted by extremely hot summers, which are characterized by rising temperatures above the thermoneutral range of pigs; as a result, they are exposed to heat stress. One of the most harmful consequences of perpetual heat stress is oxidative damage derived from the increasing level of reactive oxygen species (ROS) [1]. Heat is an important environmental stress factor that may affect to the reproductive performance of animals, in particular during summer months both in temperate [2,3] and tropical [4,5] environments. Gestation and lactation are both critical periods in which sows are more susceptible to heat stress. Several studies have reported reproductive problems in sows under high summer ambient temperature conditions, including impaired embryonic development [6]; reduced farrowing rate [7,8]; decreased litter size and increased interval between weaning to estrus [5,9,10]; and reduced feed intake and milk yield in sows [6,11]. These reproductive problems cause a decline in the productivity of pigs, which results in economic losses [12].

The oxidative damage can be evidenced by increased plasma concentration of thiobarbituric acid-reactive substances (TBARS) and protein carbonyls (CARB) [13]. Phenolic compounds are products of plant secondary metabolism, having a benzene ring with one (phenol) or more (polyphenol) hydroxyl group as esters, methyl esters etc. [14,15]. Most of the phytogenic feed additives (PFAs) based on plant extracts have beneficial antimicrobial, antioxidative, and growth-promoting effects in swine, including sows, weaning piglets, and finishers [16,17,18,19,20]. The use of PFAs substances (spices, herbs, or extracts) as feed supplements has been reported to improve certain growth and health parameters in pigs [21]. The basic mode of action of phenolic compounds as anti-oxidative agents is related to the reduction of hydrogen or electron donation that makes these compounds free-radical scavengers (antioxidants) [22]. Although the beneficial effects of PFAs have been previously reported, their effect on heat stress has been assessed in a limited number of cases [1].

The aim of this study was to evaluate the effects of PFAs on the concentration of TBARS and the protein carbonyls as biomarkers of oxidative damage, as well as on the performance of sows exposed to heat stress under field conditions.

## 2. Materials and Methods

### 2.1. Trial Farm

This study was conducted in a farrow-to-finish pig farm, with a genetic background of commercial hybrids of Large White x Landrace (DanBred). The capacity of the farm was 550 sows under production, located in Larissa (39°31′39.6″ N 22°30′41.3″ E Thessaly, Central Greece) in July 2021. The location is selected because Larissa is a region in Greece where some of the warmest temperatures in the country are usually encountered. This is translated into more days with high temperatures (maximum > 35 °C) and night temperatures exceeding 20 °C (more than 20 hot days per year) [23].

All gilts/sows had individual ear-tags and were housed in a separate mating-pregnancy building. The first service of the gilts was applied during their second estrus. The farm applied artificial insemination, using purchased semen doses from a boar stub (Duroc breed). One week prior to farrowing, the sows were moved from the mating-pregnancy building to the farrowing building in groups (16 sows per group) in order to fill one farrowing room. All farrowing rooms included pens with commercial farrowing crates, that were equipped with nipple drinkers and separate removable feeders for the sows and the piglets. No enrichment material (e.g., straw) was used in sows before and after parturition. After the weaning, sows were moved to the mating-pregnancy building and were penned separately in individual cages with slatted floors until artificial insemination (weaning to estrus interval).

All sows received a different type of feed during gestation and lactation (Table 1). The feed was home-mixed, and meal-based (depending on the environmental conditions of each season) on corn/barley/wheat–soybean. For example, during lactation period, the feed amounting to 6 kg was provided in three meals during the summer months. The diet of suckling piglets included part commercial creep feed and part liquid milk supplement. The analysis of creep feed was the following: crude protein 16.5%; crude fat 7.0%; crude ashes 5.5%; crude fiber 0.2%; lactose 8.5%; Ca 0.6%; P 0.5%; lysine 1.41%; methionine 0.48%; ileal digestible lysine 1.28%; ileal digestible methionine + cystine 0.74%; ileal digestible threonine 0.90%; Vit. A 15.500 IU/kg; Vit. D3 2.100 IU/kg; Vit. E 200 mg/kg. The synthesis of the milk replacement included crude protein 21.5%; crude fat 12.5%; crude fibre 0.05%; crude ash 7.50%; calcium 0.82%; phosphorous 0.73%; Vit. A 25.000 IU; Vit. D3 5.000 IU; Vit. E 225 mg; pucoferm (lactic acid bacteria) 1.00 × 10^9^ KBE; BioPlus 2B 1.28 × 10^9^ KBE; lysine 1.80%, methionine 0.58%; methionine + cysteine 1%; threonine 1%; tryptophane 0.34%.

In the farrowing crates there was a nipple per sow and a nipple for piglets, while the drinking water was provided automatically. An everyday check for the flow of the nipples and a monthly monitoring of the water for chemical and microbiological properties were applied

Housing facilities had a fully automated feeding system and a climate monitoring system (Argos S, Microfan B.V., Nederweert, The Netherlands). This climate monitoring system, especially for farrowing rooms, is a standalone management system per room, recording the temperature and humidity values.

The vaccination scheme of breeding stock included vaccinations against porcine reproductive and respiratory syndrome; porcine circovirus 2; Aujeszky’s disease; swine influenza; parvovirus; *Erysipelothrix rhusiopathiae;* atrophic rhinitis; *Escherichia coli;* and *Clostridium perfringens*. All breeding females were treated with a single Ivermectin injection 14 days prior to farrowing.

### 2.2. Experimental Material

The following two commercial phenolic PFAs (Life Circle Nutrition AG, Hämmerli 2d, 8855, Wangen SZ, Switzerland) were tested:

(a) commercial phenolic PFA-1 (Herb-All Heat-A): a mix of pure plants mainly composed by *Emblica officinalis; Foeniculum vulgare; Citrus sinensis;* and nut fiber (part of hickory nuts). Composition: crude fiber 14.0%; crude protein 7.1%; crude fat 2.8%; crude ash 8.2%; sodium 0.02%; lysine 0.2%; methionine 0.1%;
(b) commercial phenolic PFA-2 (Herb-All Heat-D): a mix of pure plants mainly composed by *Andrographis paniculate; Glycyrrhizia glabra; Tinospora cordifolia;* and nut fiber (part of hickory nuts). Composition: crude fiber 13.5%; crude protein 6.5%; crude fat 2.5%; crude ash 8.5%; sodium 0.02%; lysine 0.2%; methionine 0.1%.

The content of polyphenols in the above feed additives is 7.8% of GAE (gallic acid equivalents) and the ORAC (Oxygen Radical Absorbance Capacity) value is around 80,000 (umol TE/100 g). These two natural feed additives are completely based on pure plants only (no extracts) and were distributed as top-dressing (5 g per sow, once per day in the morning meals per sow).

### 2.3. Experimental Design

**Figure 1:**
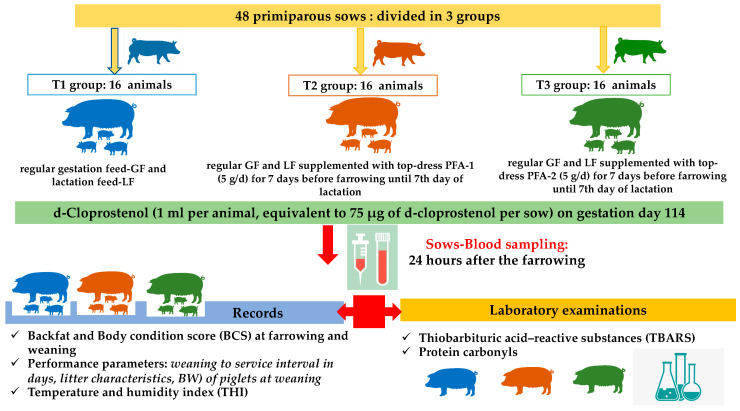
An overview of experimental design, records, samplings, and tests performed.

A total of forty-eight (48) primiparous sows of a single batch were randomly allocated to one of three groups, as shown in Figure 1:

(a) T1 group-control group (16 sows): regular gestation (GF) and lactation feed (LF);
(b) T2 group (16 sows): regular GF and LF supplemented with top-dress of the commercial phenolic PFA-1 (Herb-All Heat-A) (5 g/day) for 7 days before farrowing until 7th day of lactation;
(c) T3 group (16 sows): regular GF and LF supplemented with top-dress of the commercial phenolic PFA-2 (Herb-All Heat-D) (5 g/day) for 7 days before farrowing until 7th day of lactation.

At admission, the ear tags of the sows were recorded, and no cross fostering was allowed. Sows of all groups were housed in the same room or in identical rooms. All primiparous sows (*n* = 48) received a single injection of D-cloprostenol (1 mL per animal, equivalent to 75 μg of D-cloprostenol per animal/Gestavet Prost^®^, Hipra, Amer, Girona, Spain) at 14.00–16.00 on gestation day 114. Sows that had not farrowed by 05:30 the following day received 10 IU oxytocin. The above scheme was performed to ensure the same farrowing day for all sows of the trial, to increase the likelihood of piglet delivery during working hours as well as to allow a closer management of trial.

### 2.4. Blood Sampling

Blood samples were collected via jugular venipuncture from five primiparous sows per group, restrained by snout snare, 24 h after the farrowing. Τhe selected primiparous sows for blood sampling delivered mummies and stillborn piglets in their litters. Blood was collected 1–3 h after their first meal, using S-Monovette^®^ 9 mL, Lithium-Heparin (Sarstedt AG & Co. KG, Nümbrecht, Germany) and disposable 14Gx3-1/414, 2.1 × 80 mm needles (Jørgen Kruuse A/S, Langeskov, Denmark). Plasma samples were obtained by centrifugation (5810 R, Eppendorf AG, Hamburg, Germany) at 3000× *g* for 15 min, at 4 °C, then the supernatant was transferred into 1.5 mL microcentrifuge tubes and stored at −80 °C pending laboratory analysis.

### 2.5. Laboratory Examinations

#### 2.5.1. TBARS

For the TBARS determination, a slightly modified assay of Keles et al. [24] was employed. Briefly, 100 μL of plasma was mixed with 500 μL of 35% TCA and 500 μL of Tris–HCl (200 mmol/L; pH 7.4) and incubated for 10 min at about 20 °C.

One milliliter of 2 mol/L Na_2_SO_4_ and 55 mmol/L thiobarbituric acid solution was added, and the samples were incubated at 95 °C for 45 min. The samples were cooled on ice for 5 min and were vortexed after 1 mL of 70% TCA was added. Consequently, the mixtures were centrifuged at 15,000× *g* for 3 min, and the absorbance of the supernatant was read at 530 nm. A baseline shift in absorbance was considered by running a blank along with all samples during the measurement. Calculation of the TBARS concentration was based on the molar extinction coefficient of malondialdehyde (MDA).

#### 2.5.2. CARBs

CARBs were determined based on a previously described method [25]. Briefly, 50 μL of 20% TCA were added to 50 μL of plasma and this mixture was incubated in an ice bath for 15 min and centrifuged at 15,000× *g* for 5 min at 4 °C. The supernatant was discarded and 500 μL of 10 mmol/L 2, 4-dinitrophenylhydrazine (DNPH) in 2.5 N HCL for the sample (500 μL of 2.5 N HCL for the blank) was added in the pellet. The samples were incubated in the dark at about 20 °C. for 1 h, with intermittent vortexing every 15 min and were centrifuged at 15,000× *g* for 5 min at 4 °C. The supernatant was discarded and 1 mL of 10% TCA was added vortexed, and centrifuged at 15,000× *g* for 5 min at 4 °C.

The supernatant was discarded and 1 mL of ethanol-ethyl acetate (1:1 *v*/*v*) was added, vortexed and centrifuged at 15,000× *g* for 5 min at 4 °C. This washing step was repeated twice. The supernatant was discarded and 1 mL of 5 mol/L urea (pH 2.3) was added, vortexed and incubated at 37 °C for 15 min. The samples were centrifuged at 15,000× *g* for 3 min at 4 °C and the absorbance was read at 375 nm. Calculation of CARB concentration was based on the molar extinction coefficient of DNPH.

### 2.6. Recorded Parameters

#### 2.6.1. Sow Body Condition Parameters

The body condition of the primiparous sows was assessed both visually and by means of backfat measurements on farrowing and weaning days. Before application the backfat measurements, the sow’s body condition score (BCS) was assessed by the same person visually with a score scale of 1 to 5 [26]. Score 1 was recorded for extremely thin sows and score 5 for extremely fat ones. Backfat measurements were performed at the P2 position that is located at left side of the 10th rib and in a distance of 6 cm from the spine using pulse ultrasound (Lean-Meater^®^ Series 12, serial number 63597, Renco Corporation, Minneapolis, MN, USA). The point of measurement was marked on each sow to guarantee that the same spot was assessed during the subsequent measurements.

#### 2.6.2. Reproductive Parameters and Litter Characteristics

Reproductive parameters (weaning to estrus interval in days) and litter characteristics (number of totally born; liveborn; stillborn; mummies; alive piglets 24 h after their birth-alive >24 h; and weaning piglets), as well as BW of weaning piglets were recorded for each group.

#### 2.6.3. Thermal Comfort Indices

The indoor thermal environment was monitored hourly using data loggers (Argos S, Microfan B.V., Nederweert, The Netherlands) to measure temperature and relative humidity. The temperature and humidity index (THI) were estimated using the hourly findings of the climate control system of each farrowing room according to the following models:

(a) THI-1 = 0.8 × T + ((RH (T − 14.4)/100)) + 46.4. The threshold zone of the index THI-1 is: THI-1 < 74 for suitable environmental; 74 ≤ THI-1 < 78 means for heat stress; 78 ≤ THI2 < 82 for moderate heat stress; THI-1 > 82 for severe heat stress [22,27,28];
(b) THI-2 = (1.8 × T + 32) − (0.55 × (RH/100)) × (1.8 × T + 32) − 58 [29,30,31]. The threshold zone of the index THI-2 is: THI-2 ≤ 74 for suitable environmental; 74 < THI-2 ≤ 78 for mild heat stress; 78 < THI-2 ≤ 84 for moderate heat stress; THI-2 > 84 for severe heat stress [30,31].

### 2.7. Statistical Analysis

The results were statistically analyzed using MedCalc Statistical Software version 14.8.1 (MedCalc Software bvba, Ostend, Belgium). The normality of the data was evaluated with Shapiro–Wilk test and the homogeneity of variances was evaluated with Levene’s test. The results of the reproductive indicators, TBARS and CARB in plasma, as well as the THI indexes, were analyzed using Kruskal–Wallis test. If the Kruskal–Wallis test is positive (P less than the selected significance level) then MedCalc performs a test for pairwise comparison of subgroups, according to Conover, 1999 [32,33,34,35,36,37,38]. The correlation between the two indices of THI-1 and THI-2 was determined using Rank correlation. All comparisons were performed at a significance level of *p* < 0.05.

## 3. Results

### 3.1. Thermal Comfort Indices

**Figure 2:**
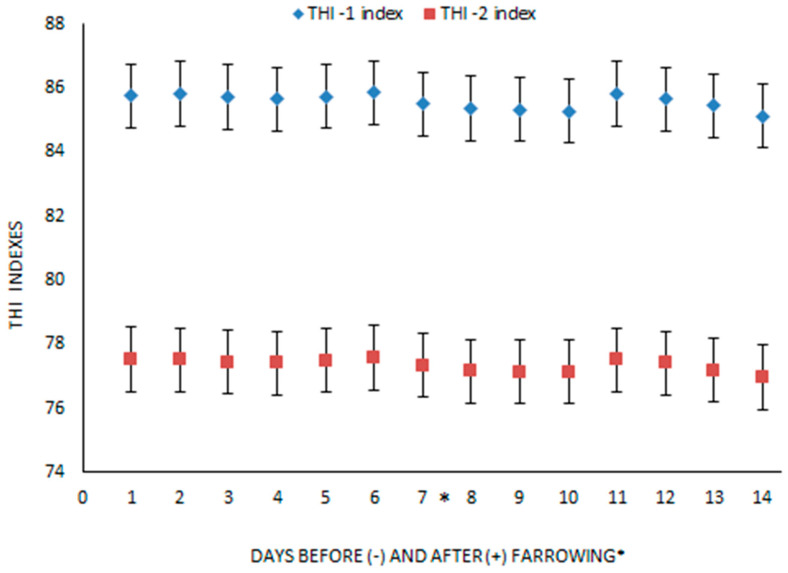
Mean daily value of THI-1 and THI-2 indexes and standard error (bars) 7 days before (-) and 7 days after (+) farrowing (*).

The mean values of the two indices of each day, for 7 days before and 7 days after farrowing, are presented in Figure 2 (the data are available Table S1). The graphical presentation shows that both daily mean THI- indices were over 74, which is the cut off value indicative of heat stress.

### 3.2. Reproductive Parameters and Litter Characteristics

**Figure 3:**
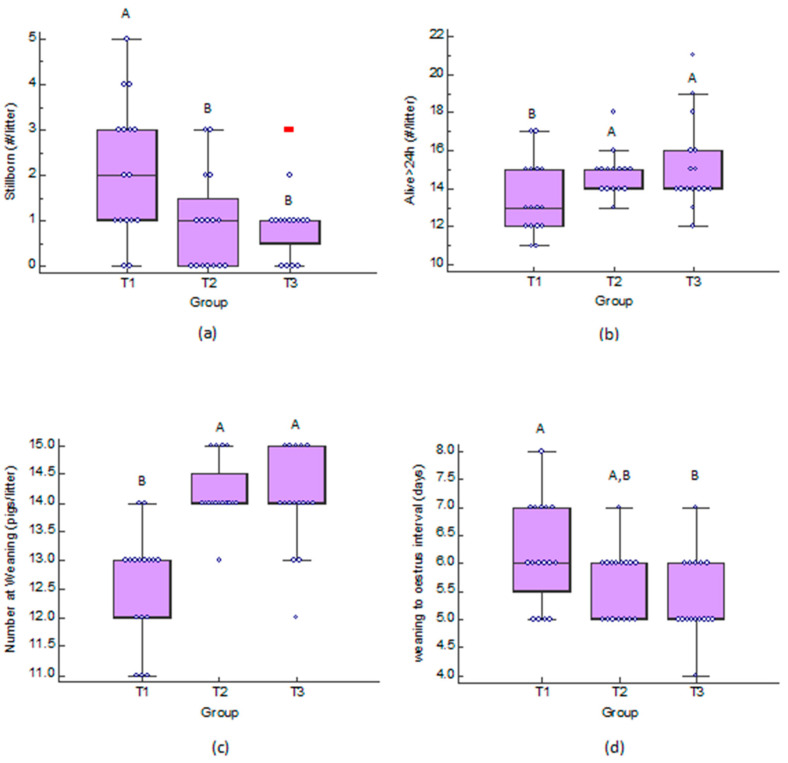
Dot and box and whisker plots of: (**a**) stillborn piglets (#/litter); (**b**) alive over 24 h piglets (#/litter); (**c**) number of weaning (pigs/litter); and (**d**) weaning to estrus interval (days), in control group (T1), group fed with PFA-1 (T2) and group fed with PFA-2 (T3). #: Number; ^A,B^ Different letters above bars are indicative of significant difference (*p* < 0.05).

A significant difference was observed in the number of stillborn piglets among the control group and the other two groups, while the highest median was found in the control group (Figure 3a). Moreover, a significant difference in the number of the piglets that remained alive after the first 24 h of their life (*p* = 0.04), as well as in the number of the weaning piglets (*p* < 0.01) was observed among the groups, while the median of the control group was found to be the lowest (Figure 3b,c). A significant difference was also observed between the control group (T1) and the T3 group, when the interval from weaning to estrus was compared between the groups (*p* = 0.03), with the control group having the highest median (Figure 3d). The data are available in S3 of the Supplementary File.

### 3.3. Sow Body Condition Parameters

**Figure 4:**
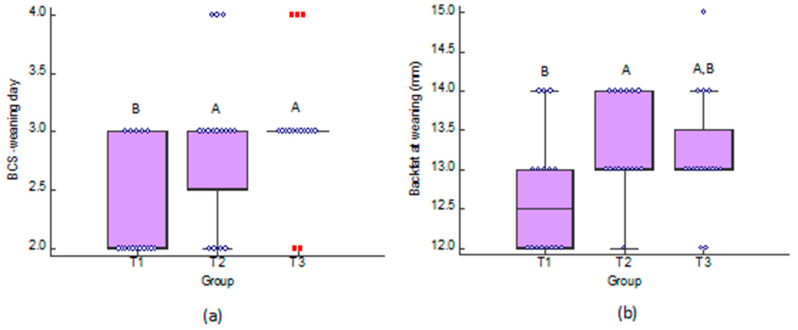
Dot and box and whisker plots of: (**a**) body condition scoring (BCS) of sows at weaning day; and (**b**) the weaning to estrus interval, in control group (T1), group fed with Herb-All HEAT-A (T2) and group fed with Herb-All HEAT-D (T3). ^A,B^ Different letters above bars are indicative of significant difference (*p* < 0.05).

A significant difference was observed in the BCS (*p* = 0.02) and the backfat (*p* = 0.04) at the weaning among the groups, while the lowest medians of the two parameters were observed in the control group one (Figure 4). The data are available in S4 of the Supplementary File.

### 3.4. Laboratory Examinations

**Figure 5:**
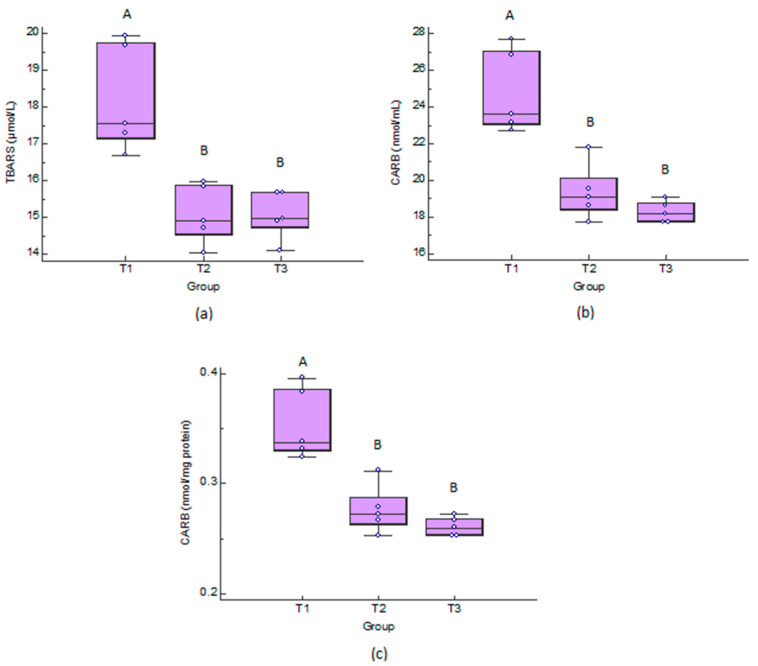
Dot and box and whisker plots of: (**a**) Thiobarbituric acid–reactive substances (TBARS); and (**b**,**c**) protein carbonyls (CARB) concentrations in control group (T1), group fed with Herb-All HEAT-A (T2) and group fed with Herb-All HEAT-D (T3). ^A,B^ Different letters above bars are indicative of significant difference (*p* < 0.05).

As is shown in Figure 5, there is a significant difference between control group and the other two groups, in the TBARS and CARB concentrations, while the highest medians were observed in the control group. The data are available in S5 of the Supplementary File.

## 4. Discussion

During our trial, the pregnant sows suffered from severe heat stress, as is shown in Figure 2. In the present study the most common indices have been used in order to better verify heat stress. Moreover, the strength of correlation was tested to determine if the two indices can be used interchangeably, in future studies. Heat stress is an important environmental stress factor on the reproductive performance of animals, especially during the summer period [2,3,4,5]. In particular, heat stress has negative effects on the overall reproductive performance of sows, such as embryonic development [6]; farrowing rate [7,8]; litter size (mainly number of live born piglets per litter); interval between weaning and the next litter [5,9,39,40,41]; as well as feed intake and milk yield in sows [6,11]. In our study, the use of phenolic PFAs in sow gestation and lactation diets had beneficial effects on their reproductive performance and litter characteristics, evidenced by the decreased number of stillborn piglets and the increased number of alive piglets 24 h after their birth. Previous studies reported a reduction of about one piglet per litter due to summer heat stress [39,40,41], as well as the beneficial effects of PFAs use in sows before farrowing to reduce the number of stillborn piglets [42]. Moreover, for piglets that are born from sows that suffered from heat stress during pregnancy, this can lead to alterations of metabolism, resulting in decreased skeletal muscle [43] and weaning BW [44]. The increased number of weaning piglets in groups of our study that received phenolic PFAs was possibly due to the improvement of feed intake and milk production of sows, and not only on the increased number of alive piglets 24 h after their birth. Heat stress has a significant negative impact on the reproductive performance of sows including reduced feed intake and milk production in sows, which result in productivity losses [6,11,45]. Sows under a high thermal environment had increased oxidative stress during late gestation, indicating that increased oxidative damage to lipid, protein, and DNA could be one of the contributing factors for the reduced reproductive performance of sows in this environment [46]. Previous studies reported that the use of PFAs in sows during the seven days before farrowing is efficient in reducing oxidative stress (e.g., the reduction of TBARS in sow serum after farrowing), improving feed intake during lactation, as well as the quality and quantity of colostrum and milk [42,47,48,49].

Highly prolific sows are characterized by a high reproductive trait, resulting in an increased metabolic heat production, which renders them more susceptible to heat stress [50,51]. Previous studies reported severe catabolic status and increased oxidative stress levels in sows during late gestation and lactation [52,53]. Based on our results, the increased BCS and backfat at weaning and the decreased weaning to estrus interval, are indications that the use of commercial phenolic PFAs could lead to an improved body condition and reproductive parameters which needs further investigation, such as feed intake measurements during lactation that are not provided in the present study.

Recent studies have shown that higher backfat thickness in sows is associated with enhanced oxidative stress, increased expression of pro-inflammatory cytokines and inhibition of a healthy placenta development observed in sows with moderate backfat thickness. Levels of ROS and MDA, a lipid peroxidation marker, were increased in the placenta of the sows with increased backfat thickness [54]. This may also affect fetal development, as lipid oxidation can influence placental development, lipid metabolism and transport. The above stresses the importance of closely controlling body condition in sows.

Heat stress has been reported to induce oxidative stress during the summer in livestock animals [55,56]. Previous studies have reported that heat stress enhances ROS production and induces oxidative stress, which can lead to cytotoxic damages [57,58]. Oxidative stress resulting from increased production of ROS, and/or a decrease in antioxidant defense, leads to damage of biological macromolecules and disruption of the normal metabolism and physiology [55,59]. Accumulation of ROS results in oxidative damage to lipids, proteins and DNA in cells and consequently tissue damage. Μοreover, the oxidative stress could be one of the stress responses caused by a high thermal environment [46] and it can lead to an increase in TBARS [13], resulting in cytotoxic effects [60]. In addition, the plasma CARΒ concentration increases during late gestation in sows under heat stress conditions, indicating that sows suffer severe oxidative damage [46]. The results of the current study indicated that the tested phenolic PFAs have strong natural antioxidant activity in sows during the late gestation, as they exhibited decreased oxidative stress-induced damage to lipids and proteins, as evidenced by the decreases in TBARS and CARB, respectively, in groups that received phenolic PFAs. Previous studies reported that phenolic PFAs enhanced the resistance of weaning pigs to stress [61] as well as the growth performance and oxidative stability of meat in farm animals [62]. Similar results of decreased TBARS and CARB after the use of a polyphenolic byproduct from olive mill wastewater were noticed in piglets, broiler chickens, and lambs [63,64,65].

The Mediterranean region suffers from climate change particularly due to its sensitivity to drought and increased temperatures [66,67,68]. These changes may adversely affect agriculture and livestock production. In Greece, climate change is expected to result in warmer temperatures, with more days with an increased temperature > 35 °C and night temperatures up to 20 °C (> 50 days annually in most areas) [69]. For instance, regions in Thessaly (where the current trial was carried out) are expected to experience up to 20 more hot days, and almost an additional month with night-time temperatures higher than 20 °C [23]. According to recent reports, by the end of the 21st century the temperature in Greece will increase significantly, expecting heat wave days (temperatures > 35 °C) to increase by 15–20 annually by 2050 [68,70,71]. A variety of PFAs were tested for their antimicrobial and growth-promoting effects in swine [16,17,18,20]. Therefore, especially in Mediterranean countries, future studies with PFAs in swine are required, not only as growth promoters or alternatives to antibiotics, but also as an efficient tool to manage the negative consequences of heat stress due to the climate change.

## 5. Conclusions

In conclusion, the use of phenolic PFAs derived from pure plants of current study has beneficial effects on primiparous sows under field heat stress conditions including:

(a) antioxidative effects (decrease of TBARS and CARB in plasma) (a) TBARS: 15.09% lower in T2 than T1; 14.70% lower in T3 than T1; (b) CARB (nmol/mL): 19.25% lower in T2 than T1; 23.00% lower in T3 than T1; (c) CARB (nmol/mg protein): 20.58% lower in T2 than T1; 23.52% lower in T3 than T1;
(b) improved reproductive parameters and litter characteristics e.g., a decrease of the interval from weaning to estrus (5 days in T3 group in comparison to control group T1), decrease of stillborn piglets;
(c) improved body condition parameters.

## Figures and Tables

**Table 1:** Composition (kg) and calculated analysis (%) of gestation feed (GF) and lactation feed (LF) of sows’ diet.

**Composition (Ingredients) (kg)**	**GF**	LF *
Corn	300	353
Barley	280	200
Wheat bran	240	200
Soybean	120	170
Soybean oil	10	20
Protein concentrate **	12.50	25
Premix of vitamins/minerals ***	30	40
Inactive yeast ****	5	5
Toxin binder *****	3	3
Powdered cellulose ****** Total	5 1005.5	5 1021
**Analysis (%)**	**GF**	LF *
Crude protein	16.50	18.40
Crude fat	3.70	4.65
Crude fiber	5.00	4.70
Lysine	0.80	0.96
Methionine	0.29	0.33
Methionine + Cystine	0.60	0.63
Calcium	0.65	0.86
Total phosphorus	0.76	0.78
Available phosphorus	0.40	0.46
Sodium	0.24	0.24

* 1st day of farrowing until weaning. ** Apsaprotein F68 (Andres Pintaluba SA, Reus, Spain). *** The source and composition of the vitamin and mineral premix is analytically presented in the Supplementary File S1. **** Prosol Expert (Prosol SPA, Madone BG, Italy). ***** Apsa Quimitox (Andres Pintaluba SA, Reus, Spain; bentonites, sepiolitic clay, dried yeast-*Sacharomyces cerevisiae*, purified diatomaceous earth). ****** Arbocel^®^ (J. Rettenmaier and Söhne GmbH, Rosenberg, Germany).

## Data Availability

The data presented in this study are available on request from the corresponding author. The data are not publicly available due to further processing for other studies.

## Supplementary Materials

The following supporting information can be downloaded at: https://www.mdpi.com/article/10.3390/antiox11030593/s1, S1 (Footnote): Premix of vitamins/minerals; Table S1: Data of THI-1 and THI-2 indexes; Table S2: Mean, standard error (SE), median, interquartile range (IQR) and *p* value of the reproductive parameters and litter characteristics and comparison between the groups; Table S3: Mean, standard error (SE), median, interquartile range (IQR) and *p* value of sow body condition parameters and comparison between the groups; Table S4: Mean, standard error (SE), median, interquartile range (IQR) and *p* value of TBARS and CARB values and comparison between the groups.
