# Supplementary material for: Effects of Phenolic Phytogenic Feed Additives on Certain Oxidative Damage Biomarkers and the Performance of Primiparous Sows Exposed to Heat Stress under Field Conditions
Source: Antioxidants (Basel). 2022 Mar 20;11(3):593. doi: 10.3390/antiox11030593 (PMC8945155; doi:10.3390/antiox11030593)
Supplement: Supplementary file 1 [file antioxidants-11-00593-s001.zip › S1.pdf]

S1 (Footnote): Premix of vitamins/minerals

GF per 30 Kg: Vit. A (3a672a) 12,000,000 iu, Vit. D_3 (3a671) 2,400,00, Vit. E (3α700) 100,000 mg, Vit. K (3a711) 2,000 mg, Vit. B_1 (3a821) 2,000 mg, Vit. B_2 5,000 mg, Vit. B_6 (3α831) 6,000 mg, Vit. B_{12} (3α315) 25 mg, Vit. B_3 (3α315) 40,000 mg, Vit. B_5 (3a841) 15,000 mg, folic acid (3α316) 4,000 mg, biotin (3α880) 300 mg, Vit. C (3α312) 105,000 mg, 3-phytase (4a1600) 500,000 FTU, 1,4-beta-xylanase 560,000 TXU, endo-β-1,4-glucanase 250,000 TGU, choline chloride (3α890) 360.000 mg, Mn(E5, manganese oxide) 60.000 mg, manganese chelate of glycine hydrate (E5) 27,200 mg, copper (3b405, copper sulphate pentahydrate) 20,000 mg, iron chelate of glycine hydrate (3b108) 12,000 mg, zinc oxide (3b603) 110,000 mg, zinc chelate of glycine hydrate (3b607) 30,000 mg, selenium (3b801, sodium selenite) 300 mg, iodine (3b202) 2,000 mg, BHT(E321) 4,500 mg, BHA(E320) 2,970 mg, E310 30 mg, E330 1, 500 mg

LF per 40 Kg: Vit. A (3a672a) 12,000,000 iu, Vit. D_3 (3a671) 2,400,00, Vit. E (3α700) 125,000 mg, Vit. K (3a711) 2,000 mg, Vit. B_1 (3a821) 2,000 mg, Vit. B_2 5,000 mg, Vit. B_6 (3α831) 6,000 mg, Vit. B_{12} (3α315) 25 mg, Vit. B_3 (3α315) 40,000 mg, Vit. B_5 (3a841) 15,000 mg, folic acid (3α316) 4,000 mg, biotin (3α880) 300 mg, Vit. C (3α312) 105,000 mg, β-carotene 5,000 mg, 1,4-beta-xylanase 560,000 TXU, endo-β-1,4-glucanase 250,000 TGU, choline chloride (3α890) 360.000 mg, Mn (E5, manganese oxide) 60.000 mg, manganese chelate of glycine hydrate (E5) 14,880 mg, copper (3b405, copper sulphate pentahydrate) 20,000 mg, copper chelate of glycine hydrate (3b413) 28,160, iron (33b10, iron sulphate monohydrate) 150,000 mg, iron chelate of glycine hydrate (3b108) 12,000 mg, zinc oxide (3b603) 110,000 mg, zinc chelate of glycine hydrate (3b607) 30,160 mg, selenium (3b801, sodium selenite) 300 mg, iodine (3b202) 2,000 mg, BHT(E321) 4,500 mg, BHA(E320) 2,970 mg, E310 30 mg, E330 1, 500 mg
