# Supplementary material for: Effects of Phenolic Phytogenic Feed Additives on Certain Oxidative Damage Biomarkers and the Performance of Primiparous Sows Exposed to Heat Stress under Field Conditions
Source: Antioxidants (Basel). 2022 Mar 20;11(3):593. doi: 10.3390/antiox11030593 (PMC8945155; doi:10.3390/antiox11030593)
Supplement: Supplementary file 1 [file antioxidants-11-00593-s001.zip › Table S2.pdf]

Table S2: Mean, standard error (SE), median, interquartile range (IQR) and *p* value of the reproductive parameters and litter characteristics and comparison between the groups.

Reproductive indicator	Group	N	Mean	SE	Median	IQR	*p* value
**Total born**	T1	16	16.56	0.86	16.50	13.00- 20.00	0.95
	T2	16	15.88	0.38	15.50	15.00- 17.00	
	T3	16	16.56	0.79	15.50	15.00- 17.50	
**Born alive**	T1	16	15.62	0.67	16.50	13.00- 18.00	0.99
	T2	16	15.43	0.32	15.00	14.50- 16.50	
	T3	16	15.88	0.64	15.00	14.00- 16.50	
**Stillborn**	T1	16	2.13	0.38	2.00 [a]	1.00- 3.00	0.03
	T2	16	0.94	0.27	1.00 [b]	0.00- 1.50	
	T3	16	1.06	0.23	1.00 [b]	0.50- 1.00	
**Mummies**	T1	16	0.75	0.23	0.50	0.00- 1.00	0.97
	T2	16	0.56	0.13	1.00	0.00- 1.00	
	T3	16	0.63	0.18	0.50	0.00- 1.00	
**Alive >24h**	T1	16	13.50	0.48	13.00 [b]	12.00- 15.00	0.04
	T2	16	14.94	0.31	15.00 [a]	14.00- 15.00	
	T3	16	15.19	0.59	14.00 [a]	14.00- 16.00	
**Weaning**	T1	16	12.56	0.24	13.00 [b]	12.00- 13.00	<0.01
	T2	16	14.19	0.14	14.00 [a]	14.00- 14.50	
	T3	16	14.06	0.21	14.00 [a]	14.00- 15.00	
**Mean BW of weaning**	T1	16	7.46	0.13	7.50	6.90- 7.90	0.32
	T2	16	7.64	0.16	7.50	7.20- 8.20	
	T3	16	7.75	0.12	7.65	7.35- 8.20	
**Weaning to oestrus interval**	T1	16	6.19	0.23	6.00 [a]	5.00- 7.00	0.03
	T2	16	5.63	0.15	6.00 [a,b]	5.00- 6.00	
	T3	16	5.38	0.18	5.00 [b]	5.00- 6.00	

*Figures with different superscripts are indicative of a statistically significant difference (*p* value < 0.05).
