# Supplementary material for: Effects of Phenolic Phytogenic Feed Additives on Certain Oxidative Damage Biomarkers and the Performance of Primiparous Sows Exposed to Heat Stress under Field Conditions
Source: Antioxidants (Basel). 2022 Mar 20;11(3):593. doi: 10.3390/antiox11030593 (PMC8945155; doi:10.3390/antiox11030593)
Supplement: Supplementary file 1 [file antioxidants-11-00593-s001.zip › Table S3.pdf]

Table S3: Mean, standard error (SE), median, interquartile range (IQR) and *p* value of sow body condition parameters and comparison between the groups.

Reproductive indicator	Group	N	Mean	SE	Median	IQR	*p* value
**Backfat at farrowing**	T1	16	17.31	0.25	17.00	16.50- 18.00	0.90
	T2	16	17.06	0.25	17.00	17.00- 18.00	
	T3	16	17.19	0.25	17.00	17.00- 18.00	
**Backfat at weaning**	T1	16	12.69	0.20	12.50 [b]	12.00- 13.00	0.04
	T2	16	13.31	0.15	13.00 [a]	13.00-14.00	
	T3	16	13.19	0.19	13.00 [a,b]	13.00- 13.50	
**BCS at farrowing**	T1	16	4.19	0.14	4.00	4.00- 4.50	0.15
	T2	16	3.88	0.18	4.00	3.00- 4.00	
	T3	16	3.75	0.17	4.00	3.00- 4.00	
**BCS - weaning day**	T1	16	2.31	0.12	2.00 [b]	2.00- 3.00	0.02
	T2	16	2.94	0.17	3.00 [a]	2.50- 3.00	
	T3	16	3.06	0.14	3.00 [a]	3.00- 3.00	

*Figures with different superscripts are indicative of a statistically significant difference (p value < 0.05).
