# Supplementary material for: Effects of Phenolic Phytogenic Feed Additives on Certain Oxidative Damage Biomarkers and the Performance of Primiparous Sows Exposed to Heat Stress under Field Conditions
Source: Antioxidants (Basel). 2022 Mar 20;11(3):593. doi: 10.3390/antiox11030593 (PMC8945155; doi:10.3390/antiox11030593)
Supplement: Supplementary file 1 [file antioxidants-11-00593-s001.zip › Table S4.pdf]

Table S4: Mean, standard error (SE), median, interquartile range (IQR) and *p* value of TBARS and CARB values and comparison between the groups.

Parameter	Group	N	Mean	SE	Median	IQR	*p* value
**TBARS (µmol/L)**	T1	5	18.23	0.66	17.55[a]	17.14- 19.74	0.009
	T2	5	15.09	0.36	14.90[b]	14.54- 15.86	
	T3	5	15.08	0.30	14.97[b]	14.71- 15.70	
**CARB (nmol/mL)**	T1	5	24.82	1.02	23.64[a]	23.07- 27.05	0.006
	T2	5	19.36	0.68	19.091[b]	18.41- 20.11	
	T3	5	18.27	0.27	18.18[b]	17.73- 18.75	
**CARB (nmol/mg protein)**	T1	5	0.35	0.01	0.34[a]	0.33- 0.39	0.006
	T2	5	0.28	0.01	0.27[b]	0.26- 0.29	
	T3	5	0.26	0.01	0.26[b]	0.25-0.27	

*Figures with different superscripts are indicative of a statistically significant difference (*p* value < 0.05).
